# P-2026. Quality Improvement Initiative to Safely Reduce Follow-up Blood Cultures through Diagnostic Stewardship During a National Blood Culture Bottle Shortage

**DOI:** 10.1093/ofid/ofaf695.2190

**Published:** 2026-01-11

**Authors:** Jeffrey Kubiak, Barbara Ross, Shirley Wang, Regina Wulff, David Kuang, Anne Norris, Trevona Gonsalves, Stacia Semple, Jamie Marino, Karen P Acker, Harold Horowitz, Tina Wang, Daniel A Green, Gregory Berry, Elizabeth Stone, David Liu, Cheryl Goss, Matthew Simon, Lars Westblade, Yoko Furuya, Harjot K Singh

**Affiliations:** Weill Cornell Medical Center, New York, New York; NewYork-Presbyterian Hospital, New York, NY; NYP, new york, New York; New York Presbyterian Hospital, New York, New York; NEW YORK PRESBYTERIAN, HOBOKEN, NewJersey; NewYork-Presbyterian Hospital, New York, NY; NewYork-Presbyterian Hospital, New York, NY; Weill Cornell Medical Center, New York, New York; Weill Cornell Medicine, New York, NY; Weill Cornell Medicine/New-York Presbyterian, New York, NY; New York Presbyterian-Brooklyn Methodist Hospital, Brooklyn, New York; Columbia University Irving Medical Center, New York, New York; Columbia University Medical Center, New York, NY; Columbia University Irving Medical Center, New York, New York; Columbia University Irving Medical Center, New York, New York; NewYork-Presbyterian Hospital, New York, NY; NewYork-Presbyterian Hospital, New York, NY; Weill Cornell Medicine, New York, NY; Weill Cornell Medicine, New York, NY; Columbia University Irving Medical Center, New York, New York; Weill Cornell Medicine, New York, NY

## Abstract

**Background:**

Blood cultures (BC) are a critical resource to diagnose bloodstream infections. However, over-ordering may lead to antibiotic overuse and increased hospital cost, length of stay, and contamination rates. A national blood culture bottle shortage prompted diagnostic stewardship of follow-up blood cultures (FUBC) in patients monitored for clearance of a prior positive BC, or with new/worsening symptoms after initial negative BC. We aimed to reduce the use of paired-set FUBC and increase single-set FUBC.

**Methods:**

This study was performed from 2/18/2024 to 11/23/2024 at a network of 8 academic and community hospitals in New York. Prior to stewardship interventions, clinicians were free to order any number of FUBC at a time. In August 2024 we implemented a series of iterative PDSA (PlanDoStudyAct) cycles to guide clinicians toward appropriate ordering of FUBC including educational memos, limiting the ability to order more than one BC at a time, an electronic best practice advisory to limit BC orders with a prior negative within 72 hours, and a new order panel for BC that defaulted FUBC to a single set. Our goal was a >10% reduction in paired FUBC. QI methodology assessed special cause, descriptive statistics were used to compare pre- and post-intervention characteristics and balancing measures, and data were analyzed using R software.

**Results:**

Over the study period 168,779 BC were ordered, of which 11% were from community hospitals. Demographic characteristics were similar before and after intervention. Prior to our QI project, 29% of BC were FUBC, of which 59% were paired sets. After the series of PDSA cycles there was a 39% increase in single FUBC, a 36% reduction in paired FUBC, and an overall 3.3% reduction in total blood culture bottle use (p = 0.002). There were no differences in the length of stay, in-hospital mortality, or 30-day readmission. Reduced paired FUBC usage was also seen in *S. aureus* bacteremia (44%) and candidemia (45%) (p < 0.001).

**Conclusion:**

We exceeded our goal of reducing paired FUBC through targeted clinical decision support while successfully increasing single-set FUBC. Outside of a blood culture bottle shortage, future iterative changes are needed to support the use of FUBC in *S. aureus* bacteremia and candidemia.

**Disclosures:**

Lars Westblade, PhD, Elements Materials Technology: Grant/Research Support|Hardy Diagnostics: Grant/Research Support|Melinta Therapeutics: Grant/Research Support|Selux Diagnostics: Grant/Research Support|Shionogi: Advisor/Consultant|SNIPRBIOME: Grant/Research Support

